# Structured Analgesia Pathways, Including Nurse-Initiated Approaches, for Acute Pain in Emergency Departments: A Systematic Review and Meta-Analysis

**DOI:** 10.7759/cureus.116566

**Published:** 2026-09-19

**Authors:** Mudathir Elyas Suleiman Khamees, Zawra Omer Ahmed Mohamedsaeed, Eithar Musa, Valentina Ahmed Ali Babeker, Asim Ahmed, Fatima Elshathliya Abubaker Alsiddig Mohammed

**Affiliations:** 1 Internal Medicine, Al-Zaiem Al-Azhari University, Khartoum, SDN; 2 General Practice, Tayma General Hospital, Tayma, SAU; 3 General Practice, Doha Clinic Hospital, Doha, QAT; 4 Family Medicine, Tabuk Primary Healthcare Center (PHC), Tabuk, SAU; 5 Medicine and Surgery, University of Gezira, Wad Madani, SDN; 6 Emergency Medicine, National University, Khartoum, SDN

**Keywords:** acute pain, emergency department, nurse-initiated analgesia, protocol-driven analgesia, triage analgesia

## Abstract

Acute pain is a common reason for emergency department (ED) attendance, yet delayed analgesia remains a persistent challenge in emergency care. Structured analgesia pathways, including nurse-initiated approaches, may reduce treatment delays by enabling earlier pain assessment and analgesic administration according to predefined criteria. This systematic review and meta-analysis evaluated the effects of structured analgesia pathways on acute pain management in ED settings, focusing on time to analgesia, pain reduction, analgesia administration, patient satisfaction, adverse events, and ED flow. A comprehensive database search was conducted, and eligible studies were independently screened, extracted, and appraised. Sixteen studies were included. The evidence base consisted mainly of non-randomized comparative studies, quality improvement projects, and implementation studies, and the review protocol was not prospectively registered. Meta-analysis supported a reduction in time to analgesia with structured pathways; however, results varied considerably across studies, while sensitivity analysis supported the same overall direction of effect. Overall, structured analgesia pathways were associated with earlier analgesic administration and improved pain management processes, particularly when treatment could begin at triage or before physician assessment. Findings for pain intensity, satisfaction, safety, and ED length of stay were generally favorable but less consistent because of differences in study design, outcome definitions, and reporting methods. Risk of bias was driven mainly by non-randomized implementation, residual confounding, and incomplete standardization of outcome reporting. These findings suggest that structured analgesia pathways may improve the timeliness and reliability of emergency pain management, although stronger comparative studies are needed to clarify their effects on patient-centered and system-level outcomes.

## Introduction and background

Acute pain is a common and clinically important reason for emergency department presentation, yet delayed and inadequate analgesia remains a persistent problem in emergency care. The undertreatment of acute pain, commonly described as oligoanalgesia, has been recognized for decades and continues to affect patients across different emergency settings [1-3]. Large multicenter data have shown that patients presenting with moderate or severe pain may experience substantial pain intensity and suboptimal pain management during emergency department care [2]. The consequences of inadequate acute pain management extend beyond patient comfort and may include physiological stress, impaired function, dissatisfaction with care, and potentially worse short- and long-term pain outcomes [3,4].

Timely pain assessment and analgesic administration are therefore central indicators of emergency care quality. Contemporary emergency medicine guidance emphasizes that pain should be assessed, treated promptly, and reassessed during the emergency department stay [5,6]. However, effective pain management in emergency departments remains challenging because crowding, triage priorities, diagnostic uncertainty, and competing clinical demands can delay treatment. These pressures may be particularly important for patients awaiting physician assessment, imaging, or definitive diagnosis.

Several strategies have been proposed to reduce delays in emergency department analgesia. These include analgesia protocols and standing orders, triage- or nurse-initiated pathways, clinical guidelines or directives, and electronic order sets [5-8]. The underlying rationale is that suitable patients with acute pain can be identified early and started on appropriate analgesia before completion of the traditional physician-led sequence of assessment, ordering, and medication administration. This approach is especially useful for painful conditions where initial analgesia can be safely protocolized, such as musculoskeletal injuries, renal or biliary colic, hip fracture, vaso-occlusive pain episodes, and other acute painful presentations.

Previous reviews have suggested that nurse-initiated medications and nurse-initiated analgesia can improve timeliness and may be safe and beneficial in emergency care settings [9,10]. A broader systematic review of nurse-initiated interventions also reported favorable effects on time-to-analgesia, time-to-treatment, pain relief, and selected emergency department performance outcomes [11]. These findings support the role of emergency nurses in earlier treatment delivery when clear governance, eligibility criteria, and safety safeguards are in place, allowing timely analgesia while protecting patient safety. Nevertheless, the interventions evaluated across previous literature have been heterogeneous, ranging from single-drug triage protocols to multifaceted quality-improvement programs, with outcomes reported in different formats.

Pain management improvement interventions in emergency departments have also been reviewed more broadly. Sampson et al. found that interventions including protocols, guidelines, education, pain scoring tools, and changes in nursing roles were associated with reported improvements in pain care, while also highlighting limitations related to study design, heterogeneity, and risk of bias [12]. Although the direction of evidence is promising, the magnitude and consistency of benefit remain uncertain for protocol-driven or nurse-initiated analgesia pathways and clinically measurable outcomes such as time to analgesia. The present review therefore addresses a more focused clinical question and also incorporates more recent evidence; five included studies were published in 2024-2025.

Despite growing interest in protocol-driven analgesia, several gaps remain. Previous reviews have often combined multiple nurse-initiated interventions beyond analgesia or evaluated broader pain-management improvement strategies. Many available studies also use non-randomized, pre-post, retrospective, or quality-improvement designs, making design-appropriate risk-of-bias assessment important. In addition, outcome reporting varies substantially across studies, including means or medians for time to analgesia, binary timely analgesia outcomes, pain score reduction, satisfaction, adverse events, and emergency department length of stay. These differences limit direct comparison and determine which data can reasonably support meta-analysis.

An updated and focused synthesis is therefore needed to evaluate protocol-driven, nurse-initiated, and structured analgesia pathways in emergency department settings while distinguishing outcomes suitable for quantitative synthesis from those requiring narrative interpretation.

This systematic review and meta-analysis evaluated the effect of protocol-driven, nurse-initiated, or structured analgesia pathways on acute pain management outcomes in emergency department settings. The primary objective was to determine whether these interventions reduce time to analgesia compared with usual care, physician-led analgesia, or pre-implementation practice. Secondary objectives included analgesia administration, pain score reduction, patient or caregiver satisfaction, adverse events, emergency department length of stay, pain assessment documentation, and protocol-related process outcomes. A meta-analysis was conducted where sufficient comparable data were available.

## Review

Methods

Study Design and Reporting Framework

This systematic review was conducted to evaluate whether protocol-driven, nurse-initiated, or structured analgesia pathways improve acute pain management outcomes in emergency department settings. The review was reported in accordance with the Preferred Reporting Items for Systematic Reviews and Meta-Analyses (PRISMA) 2020 statement [13]. The protocol was not registered.

Review Question and PICO Framework

The review question was: Among patients presenting to emergency departments with acute pain, do protocol-driven or nurse-initiated analgesia pathways improve time to analgesia, pain outcomes, safety, satisfaction, and emergency department flow compared with usual care, physician-led analgesia, or pre-implementation practice? The PICO framework is summarized in Table 1.

**Table 1:** PICO framework PICO framework defining the review population, interventions, comparators, and outcomes.

Element	Definition used in this review
Population	Adults, children, or mixed-age patients presenting to an emergency department or accident and emergency setting with acute pain or painful emergency conditions.
Intervention	Protocol-driven analgesia, nurse-initiated analgesia, triage-initiated analgesia, standing-order analgesia, structured pain pathways, fast-track pain protocols, medical directives, or electronic order-set analgesia pathways.
Comparator	Usual care, physician-led analgesia, non-nurse-initiated analgesia, standard pre-protocol care, historical controls, concurrent controls, or routine emergency department practice.
Outcomes	Primary outcome: time to analgesia. Secondary outcomes: pain score reduction, receipt of analgesia, analgesia within a defined time target, patient or caregiver satisfaction, adverse events, time to imaging, emergency department length of stay, documentation of pain assessment, and protocol adherence.

Eligibility Criteria

Studies were selected according to predefined eligibility criteria related to the setting, intervention, outcomes, study design, and publication type. The inclusion and exclusion criteria are summarized in Table 2.

**Table 2:** Inclusion and exclusion criteria The table summarizes the predefined inclusion and exclusion criteria used to determine study eligibility.

Type	Criterion
Inclusion	Studies conducted in an emergency department setting.
Inclusion	Studies evaluating nurse-initiated, triage-initiated, protocol-driven, or other structured analgesia interventions.
Inclusion	Studies reporting at least one relevant pain-care outcome.
Inclusion	Randomized, quasi-experimental, observational, pre-post, audit, quality-improvement, implementation, or record-review studies.
Inclusion	Studies were eligible regardless of country, publication year, or language.
Exclusion	Studies limited to chronic, postoperative, inpatient, outpatient, primary-care, or unlinked prehospital pain management.
Exclusion	Studies evaluating physician-only analgesia without a structured protocol.
Exclusion	Studies without extractable pain-care outcomes.
Exclusion	Reviews, editorials, protocols without results, commentaries, letters without primary data, and case reports.
Exclusion	Duplicate publications or overlapping study cohorts.

Information Sources and Search Strategy

A systematic search was conducted across electronic databases from inception to 24 June 2026. The databases searched included PubMed/MEDLINE, Scopus, Web of Science, Embase, CINAHL, the Cochrane Library, and Google Scholar. Reference lists of included studies and relevant reviews were also screened to identify additional eligible studies. No language restriction was applied. Trial registries, conference abstract databases, and other grey-literature sources were not searched.

The search strategy combined terms related to emergency care, pain or analgesia, and structured or nurse-initiated analgesia pathways. Search terms were adapted for each database. The exact Cochrane Library Boolean syntax used at the time of the original search was not preserved in the retained search record; therefore, the documented Cochrane search concepts are reported rather than reconstructing an exact query retrospectively. The search strategy is summarized in Table 3.

**Table 3:** Search strategy by database Search strategies used across electronic databases and supplementary sources. Abbreviations: ED, emergency department; A&E, accident and emergency; TITLE-ABS-KEY, title, abstract, and keywords; TS, topic search; /exp, exploded subject heading.

Database/source	Search strategy/documented search terms
PubMed/MEDLINE	("emergency department" OR "emergency room" OR "accident and emergency" OR ED OR A&E) AND (pain OR analgesia OR analgesic OR opioid OR fentanyl OR ketorolac) AND ("nurse initiated" OR "nurse-initiated" OR "triage initiated" OR "triage-initiated" OR "protocol-driven" OR protocol OR pathway OR guideline OR "standing order" OR "medical directive" OR "order set") AND ("time to analgesia" OR "time-to-analgesia" OR "pain score" OR "pain reduction" OR satisfaction OR "length of stay" OR safety OR "adverse events")
Scopus	TITLE-ABS-KEY(("emergency department" OR "emergency room" OR "accident and emergency") AND (pain OR analgesia OR analgesic) AND ("nurse initiated" OR "triage initiated" OR protocol OR pathway OR guideline OR "standing order" OR "medical directive") AND ("time to analgesia" OR "pain reduction" OR satisfaction OR safety OR "length of stay"))
Web of Science	TS=("emergency department" OR "emergency room" OR "accident and emergency") AND TS=(pain OR analgesia OR analgesic) AND TS=("nurse initiated" OR "triage initiated" OR protocol OR pathway OR guideline OR "standing order" OR "medical directive") AND TS=("time to analgesia" OR "pain score" OR satisfaction OR safety OR "length of stay")
Embase	('emergency department'/exp OR 'emergency room' OR 'accident and emergency') AND ('pain'/exp OR analgesia OR analgesic) AND ('nurse initiated' OR 'triage initiated' OR protocol OR pathway OR guideline OR 'standing order' OR 'medical directive') AND ('time to analgesia' OR 'pain reduction' OR satisfaction OR safety OR 'length of stay')
CINAHL	("emergency department" OR "emergency room" OR "accident and emergency") AND (pain OR analgesia OR analgesic) AND ("nurse initiated" OR "triage initiated" OR protocol OR pathway OR guideline OR "standing order" OR "medical directive") AND ("time to analgesia" OR "pain score" OR satisfaction OR safety OR "length of stay")
Cochrane Library	Documented search concepts: "emergency department", pain, analgesia, protocol, "nurse initiated", and "triage initiated". The exact executed Boolean syntax was not retained in the review record.
Google Scholar and reference lists	Targeted searches using combinations of "emergency department", "nurse-initiated analgesia", "triage analgesia", "pain protocol", "time to analgesia", and screening of reference lists from eligible articles.

Study Selection

All retrieved records were imported into a reference-management file, and duplicates were removed before screening. Two reviewers independently screened titles and abstracts against the eligibility criteria. Full texts were then retrieved for potentially eligible records and assessed independently by the same two reviewers. Disagreements were resolved through discussion, and when consensus could not be reached, a third reviewer adjudicated the decision. Reasons for exclusion at the full-text stage were recorded and summarized in the PRISMA flow diagram.

Data Extraction

Two reviewers independently extracted data using a standardized extraction form. Extracted items included study characteristics, population and setting, intervention and comparator details, sample size, analgesia timing and pain outcomes, treatment delivery, satisfaction, safety, emergency department flow outcomes, effect estimates, confidence intervals, p-values, and information required for risk-of-bias assessment.

For studies reporting multiple outcomes, time to analgesia was prioritized as the primary outcome. When studies reported medians, interquartile ranges, statistical process control outcomes, single-arm implementation data, or incomplete variance estimates, these findings were extracted for narrative synthesis but were not automatically pooled.

Data Items and Effect Measures

For continuous outcomes, extracted data included the reported group summaries, such as means with standard deviations, medians with interquartile ranges or ranges, or reported mean differences with confidence intervals, as available. For binary outcomes, event counts and total sample sizes were extracted when reported. The primary effect measure for the quantitative synthesis of time to analgesia was the mean difference in minutes.

Risk-of-Bias Assessment

Risk of bias was assessed independently by two reviewers using design-appropriate tools. RoB 2 was planned for randomized controlled trials [14], but no randomized trial met the inclusion criteria, and the tool was therefore not applied. Comparative non-randomized studies were assessed using ROBINS-I [15]. Single-arm implementation or descriptive protocol-evaluation studies without a formal comparator were assessed using the Joanna Briggs Institute critical appraisal checklist [16].

For ROBINS-I studies, the seven domains considered were bias due to confounding, selection of participants, classification of interventions, deviations from intended interventions, missing data, measurement of outcomes, and selection of the reported result. JBI-assessed single-arm studies were judged using the applicable checklist items rather than ROBINS-I domains. Disagreements were resolved by discussion between the two reviewers.

Data Synthesis

A narrative synthesis was conducted for all included studies. Studies were grouped according to intervention type, study design, population, and outcome category. Outcomes were summarized as time to analgesia, analgesia administration, pain intensity reduction, patient or caregiver satisfaction, adverse events, and emergency department flow outcomes. Because the included studies differed in design, patient population, intervention components, and outcome reporting, narrative synthesis was used for studies that were not suitable for pooling.

Meta-Analysis

A meta-analysis was conducted when at least two studies reported sufficiently comparable comparative numerical data for the same outcome. The primary meta-analysis outcome was time to analgesia, measured in minutes. The original analysis was performed in jamovi using the MAJOR module; the exact jamovi version was not preserved in the retained analysis record. For the revision, the final pooled estimates were independently reproduced from the retained study-level mean differences and standard errors in Python 3.13.5 using a DerSimonian-Laird random-effects estimator, yielding the same pooled results. The effect measure was the mean difference, calculated as the intervention or protocol-driven analgesia group minus the comparator group. A negative mean difference indicated a shorter time to analgesia in the intervention group.

Because the pooled evidence was based on a small number of non-randomized studies, the meta-analysis was interpreted as exploratory and supportive rather than as evidence of a causal effect. Random-effects pooling does not remove confounding inherent in before-after or quasi-experimental comparisons, so pooled estimates were interpreted alongside study design and risk-of-bias judgments.

For studies reporting group-level means and standard deviations, these values were extracted directly. When a study reported a mean difference with a 95% confidence interval but lacked complete group-level standard deviations, the standard error was derived from the confidence interval. The study was then entered using a generic inverse-variance approach. A random-effects model was used because clinical and methodological heterogeneity was expected. Statistical heterogeneity was assessed using I² and interpreted cautiously in relation to the number and clinical characteristics of the pooled studies. Sensitivity analysis was conducted when a study had a substantially larger effect estimate or contributed materially to heterogeneity. Studies reporting medians only, single-arm data, statistical process control results, or insufficient variance information were retained in the narrative synthesis rather than automatically pooled. For studies reporting means with usable variance estimates, the reported means were analyzed as presented; potential skewness in time-to-analgesia distributions was considered when interpreting pooled estimates. Publication-bias tests and prediction intervals were not performed because the number of pooled studies was too small for meaningful interpretation.

For binary outcomes, such as receipt of analgesia or analgesia within a specified time target, event counts and total sample sizes were extracted when available. Binary pooling was not ultimately performed because outcome definitions, time thresholds, and reporting formats were not sufficiently comparable across studies. These outcomes were therefore summarized narratively.

Certainty of Evidence

Certainty of evidence for the primary outcome, time to analgesia, was assessed using the GRADE approach. Judgments considered risk of bias, inconsistency, indirectness, imprecision, and publication bias, with particular attention to the non-randomized designs, residual confounding, and heterogeneity of the contributing studies. Certainty was categorized as high, moderate, low, or very low and summarized in a Summary-of-Findings table.

Results

Study Selection

The database search identified 970 records. After removing 233 duplicate records, 737 records remained for title and abstract screening. Of these, 671 records were excluded because they were clearly unrelated to protocol-driven analgesia, nurse-initiated analgesia, emergency department pain pathways, or eligible acute pain outcomes. Sixty-six reports were sought for full-text retrieval. Thirteen reports could not be retrieved despite attempts to access the full text, leaving 53 reports for full-text eligibility assessment. Thirty-seven reports were excluded after full-text review. The main reasons for exclusion were absence of a protocol-driven or nurse-initiated analgesia intervention, wrong clinical setting, absence of relevant extractable outcomes or comparator data, non-original article type, overlapping or duplicate cohorts, or ineligible population. Sixteen studies met the inclusion criteria and were included in the final synthesis [17-32]. The study selection process is summarized in Figure 1.

**Figure 1:**
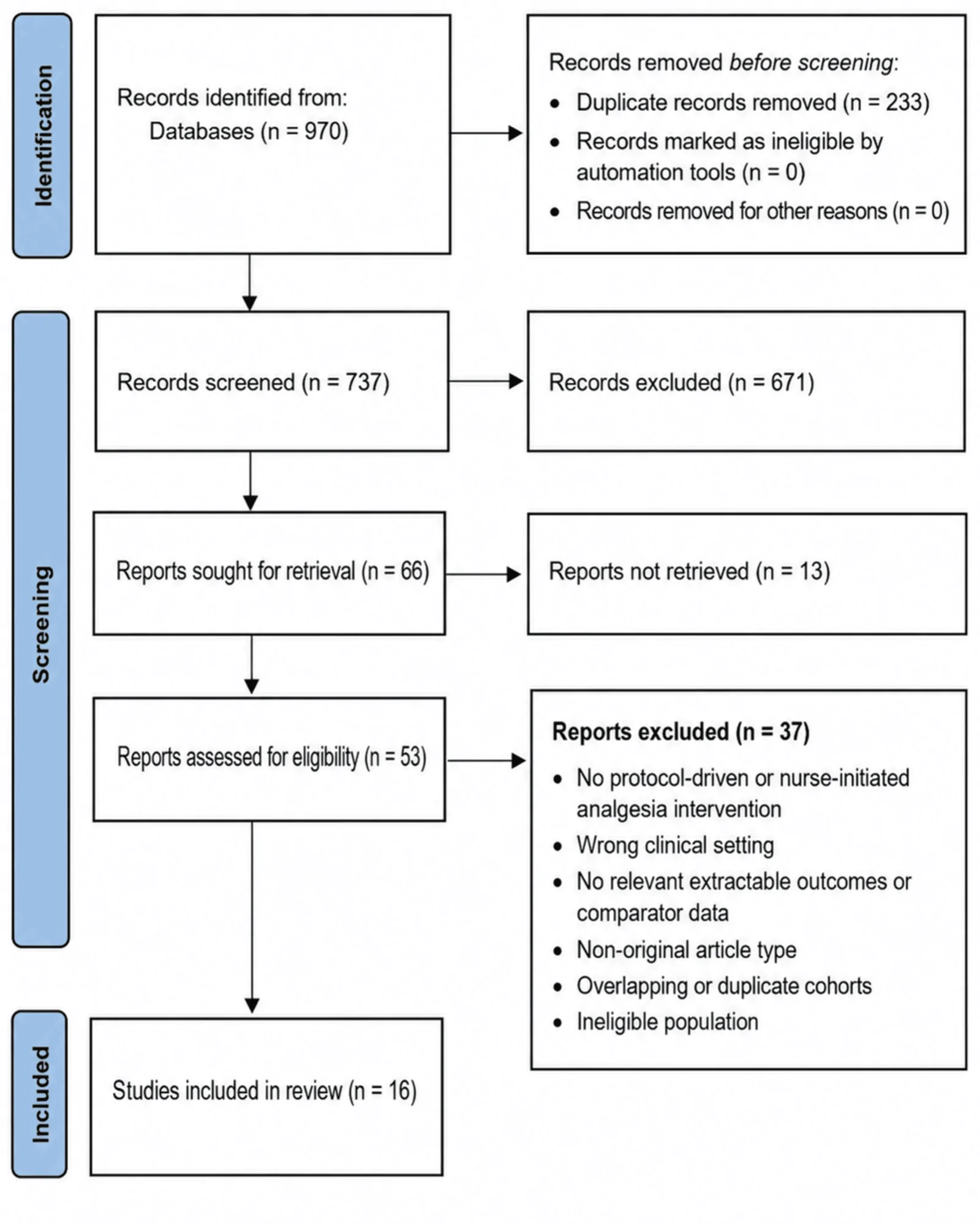
PRISMA flow diagram of study selection This figure summarizes the study selection process, including identification, screening, eligibility assessment, and final inclusion of studies in the review.

The 16 included studies were published between 1996 and 2025 and were conducted across Brazil, Australia, the Netherlands, Spain, India, Canada, Thailand, Ethiopia, Singapore, the United Kingdom, Italy, and the United States [17-32]. Most studies used non-randomized designs, including prospective cohort studies, retrospective medical record reviews, quasi-experimental studies, before-and-after implementation studies, audit and re-audit designs, and quality improvement projects. No conventional randomized controlled trial was included.

The included studies encompassed broad adult emergency department pain presentations and specific high-priority groups, including hip fracture, renal or biliary colic, musculoskeletal injuries, sickle cell vaso-occlusive crisis, and pediatric sickle cell pain episodes [18,19,22-24,31]. Interventions were similarly heterogeneous but shared a structured approach to analgesia delivery, including triage- or nurse-initiated pathways, protocol-based pain management, fast-track pathways, clinical directives, and electronic order-set care [17-32].

The most frequently reported outcomes were time to analgesia, analgesia administration, pain score reduction, patient satisfaction, adverse events, and emergency department flow outcomes such as length of stay or time to imaging. Time to analgesia was the most consistently reported outcome and therefore represented the most suitable outcome for quantitative synthesis. The main characteristics of the included studies are summarized in Table 4.

**Table 4:** Study characteristics and data extraction ED, emergency department; EMR, electronic medical record; HFFT, hip fracture fast-track; IV, intravenous; LOS, length of stay; NIA, nurse-initiated analgesia; NRS, Numeric Rating Scale; QI, quality improvement; SCD, sickle cell disease; TTA, time to analgesia.

Study	Country	Design	Population	Intervention	Comparator	Sample size	Main extracted outcomes
Santos MBD et al., 2021 [17]	Brazil	Prospective cohort	Adult ED patients with semi-urgent pain	Nurse-initiated pain protocol	Physician-first conventional care	185	Pain reduction; ED length of stay
Smith J et al., 2024 [18]	Australia	Retrospective QI review	Adult ED hip fracture patients	Triage intranasal fentanyl guideline	Historical/concurrent usual care	112	Time to analgesia; time to X-ray; ED LOS; adverse events
Admiraal M et al., 2023 [19]	Netherlands	Retrospective implementation cohort	Adults with SCD vaso-occlusive crisis	Multimodal ED pain protocol	No direct comparator	550 ED presentations	Feasibility; multimodal analgesia; time to opioid
Rodríguez-Montalvo JA et al., 2020 [20]	Spain	Retrospective protocol evaluation	Patients aged 14–65 with moderate ED pain	Advanced nursing triage oral analgesia kit	No formal comparator	400 activations	Protocol adequacy; need for additional analgesia
Patil A et al., 2017 [21]	India	Pre-post study	Adults aged 18–65 with NRS ≥4	Protocol-based ED pain management	Pre-protocol usual care	200	Time to analgesia; NRS change; satisfaction
Kelly AM et al., 2005 [22]	Australia	Retrospective record review	Adults with renal/biliary colic	Nurse-initiated titrated IV opioid	Non-nurse-initiated opioid	157 presentations	Time to first opioid; adverse events
Woolner V et al., 2020 [23]	Canada	QI pre-post project	Adult ED musculoskeletal pain	Nurse-initiated analgesia and medical directives	Baseline care	372 baseline charts; 240 TTA sample	TTA; ED LOS; analgesia administration
Sri-On J et al., 2024 [24]	Thailand	Retrospective pre-post	Older adults with hip fracture	Hip fracture fast-track protocol	Pre-HFFT care	258	Pain screening; analgesic use; regional blocks
Kefyalew M et al., 2024 [25]	Ethiopia	Quasi-experimental	ED patients with pain	Triage nurse-initiated analgesia	Routine care	179	TTA; satisfaction; LOS
Goh HK et al., 2007 [26]	Singapore	Retrospective chart review	Patients ≥16 with isolated painful limb conditions	Triage nurse ketorolac protocol	Physician analgesia after consultation	273 reviewed; 105 analgesia recipients	TTA; adverse drug reactions
Goodacre SW et al., 1996 [27]	United Kingdom	Audit/re-audit	A&E skeletal injury patients	Analgesia protocol	Pre-protocol audit	400	Analgesia use; unsatisfactory analgesia; IV opiate use
Butti L et al., 2017 [28]	Italy	Prospective observational	ED patients ≥4 years with pain	Triage nursing pain protocol	No formal comparator	382 enrolled	TTA; NRS reduction; satisfaction
Van Woerden G et al., 2016 [29]	Netherlands	Prospective pre-post cohort	Adults with ED pain NRS 4–10	Revised nurse-administered analgesia guideline	Pre-implementation care	2,107	Analgesia administration; NRS; predictors
Burgess L et al., 2025 [30]	Australia	Mixed-methods pre-post implementation	Adult ED acute pain	Evidence-based pain interventions with NIA	Pre-implementation care	Approximately 1,260 records	Analgesia within 30 min; NIA; pain documentation
Wong AF et al., 2025 [31]	USA	Pediatric QI project	Children with SCD pain requiring opioids	SCD order set and ED pathway	Baseline QI period	447 encounters	Rooming-to-analgesia; pain reassessment; ED LOS
Barksdale AN et al., 2016 [32]	USA	Retrospective pre-post EMR study	Adults with painful ED conditions	ED triage pain protocol	Pre-protocol care	23,409	Time to analgesics; protocol use

Key Findings

Across the included studies, structured analgesia pathways generally shortened time to analgesia, although absolute times varied markedly across settings. Kefyalew M et al. reported the largest reduction, from 419.9 to 88.9 minutes [25]. Goh HK et al. likewise reported substantially earlier analgesia with nurse initiation [26]. Kelly AM et al. observed a shorter median time to the first opioid dose with nurse-initiated treatment [22]. Smith J et al. also reported earlier analgesic administration after triage-initiated intranasal fentanyl in patients with hip fracture [18]. Taken together, these studies show a consistent direction toward earlier treatment, but their absolute effect sizes should not be compared directly because baseline delays, patient groups, and implementation settings differed.

Improvements in analgesia delivery and documentation were reported across several implementation studies, including higher analgesia administration after guideline implementation [29], gains in timely and nurse-initiated analgesia with better pain-score documentation [30], improved triage pain screening and analgesic use [24], and shorter time to analgesia with greater use of medical directives [23]. Collectively, these findings suggest that structured pathways improved related process measures despite differences in implementation strategy.

Pain-intensity findings were generally favorable but were measured using different scales and time points, limiting direct comparison. Patil A et al. reported greater numeric pain-score reduction after protocol implementation [21]. Butti L et al. reported a significant reduction in Numeric Rating Scale scores after triage-based analgesia [28]. Santos MBD et al. reported a greater reduction in patient-reported numeric pain scores from triage to discharge with the nurse-initiated protocol, although total emergency department length of stay was not significantly reduced [17].

Safety outcomes were reassuring where reported. Studies evaluating nurse-initiated opioid or non-opioid analgesia generally reported no major adverse events or very low adverse event rates [18,22,26,31]. However, adverse event reporting was inconsistent and was not sufficiently standardized for pooled safety analysis. Quantitative results, effect estimates, confidence intervals, and p-values are summarized in Table 5.

**Table 5:** Key quantitative findings CI, confidence interval; ED, emergency department; HFFT, Hip Fracture Fast-Track; LOS, length of stay; MD, mean difference; NIA, nurse-initiated analgesia; NRS, Numeric Rating Scale; QI, quality improvement; SPC, statistical process control; TTA, time to analgesia.

Study	Key numerical findings	p-value/CI	Direction
Santos MBD et al., 2021 [17]	ED LOS 199.2 ± 108.6 vs. 212.6 ± 91.7 min	p=0.17	No LOS benefit; numeric pain-score reduction favored protocol
Smith J et al., 2024 [18]	TTA 53 vs. 110 min; ED LOS 234 ± 59 vs. 298 ± 128 min	ED LOS MD −63.9 min, 95% CI −103.6 to −24.2; p=0.002	Favored protocol
Admiraal M et al., 2023 [19]	Multimodal treatment in 508/550 ED presentations; median time to opioid 34 min	No comparative p-value	Feasible, rapid care
Rodríguez-Montalvo JA et al., 2020 [20]	357/400 adequate activations; 217/357 required no further analgesia	60.8%, 95% CI 55.6–66.0	Favored protocol usefulness
Patil A et al., 2017 [21]	TTA: pre-protocol 20.30 vs. post-protocol 13.05 min; NRS change: pre-protocol 4.68 vs. post-protocol 5.36	TTA p<0.001; NRS p<0.001	Favored protocol
Kelly AM et al., 2005 [22]	Median TTA 31 vs. 57 min	Difference 26 min, 95% CI 16–36; p<0.0001	Favored nurse initiation
Woolner V et al., 2020 [23]	TTA 129 to 100 min; medical directive use 22% to 44%	TTA p<0.05; medical directive p<0.001	Favored intervention
Sri-On J et al., 2024 [24]	Triage pain screening 51.72% to 86.62%; analgesic use 67.24% to 78.17%	p<0.001; p=0.049	Favored HFFT
Kefyalew M et al., 2024 [25]	TTA 88.9 vs. 419.9 min	MD −331.09 min, 95% CI −446.69 to −215.5; p<0.01	Strongly favored intervention
Goh HK et al., 2007 [26]	Triage-to-analgesia 2.5 ± 8.9 vs. 68.2 ± 59.5 min	p<0.0001	Strongly favored nurse initiation
Goodacre SW et al., 1996 [27]	Unsatisfactory analgesia 91% to 69%; admissions 39% to 22%; IV opiate 9% to 37%	p<0.001; p=0.009; p<0.001	Favored protocol
Butti L et al., 2017 [28]	Mean time to analgesic intake 5.9 min; NRS reduction 2.24 at 60 min	95% CI 2.03–2.45; p<0.0001	Favored triage protocol
Van Woerden G et al., 2016 [29]	Analgesia administration 25.4% to 31.7/32.0%; adjusted OR 1.35	95% CI 1.11–1.65; p=0.001	Favored guideline
Burgess L et al., 2025 [30]	Analgesia within 30 min 21% to 27%; NIA 17% to 27%; pain score recorded 65% to 75%	p=0.032; p<0.001; p=0.017	Favored implementation
Wong AF et al., 2025 [31]	Rooming-to-analgesia 50 to 32 min; pain assessments 2.2 to 2.7	SPC/QI analysis; no conventional p-value	Favored pathway
Barksdale AN et al., 2016 [32]	Mean time to analgesics 238 to 168 min	p<0.0001	Favored triage protocol

Risk of Bias

Risk of bias was assessed according to study design. Comparative non-randomized studies were appraised with ROBINS-I, whereas the three non-comparative evaluations were appraised separately with the JBI checklist. Because no randomized controlled trials were included, RoB 2 was not applied. Detailed tool-selection methods are reported in the Methods section.

Results are presented according to the appraisal tool rather than combining different frameworks. For comparative studies, ROBINS-I judgments covered all seven standard domains and an overall judgment. The non-comparative studies were not assigned ROBINS-I categories; their JBI item-level appraisal is shown separately. The ROBINS-I domain-level assessment is shown in Figure 2.

**Figure 2:**
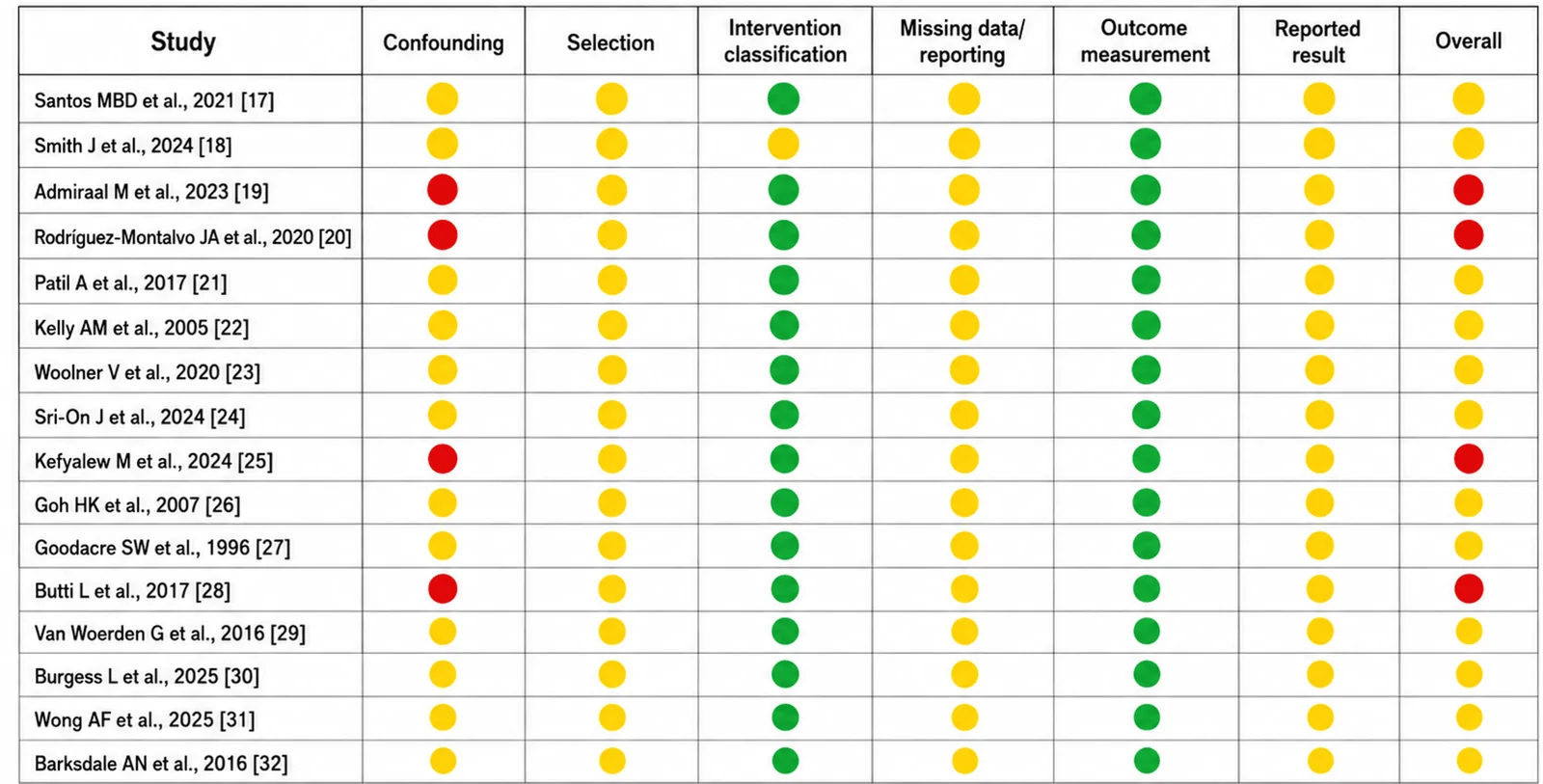
Traffic light map of risk of bias across included studies The figure presents domain-level risk-of-bias judgments for each included study. Comparative non-randomized studies were assessed using ROBINS-I, including cohort, retrospective record-review, pre-post, quasi-experimental, audit/re-audit, and quality improvement designs [17,18,21-27,29-32]. Single-arm or descriptive protocol-evaluation studies without a formal comparator were assessed using the Joanna Briggs Institute checklist [19,20,28]. Green indicates low risk of bias, yellow indicates moderate risk or some concerns, and red indicates serious risk of bias.

The overall distribution of judgments is summarized in Figure 3.

**Figure 3:**
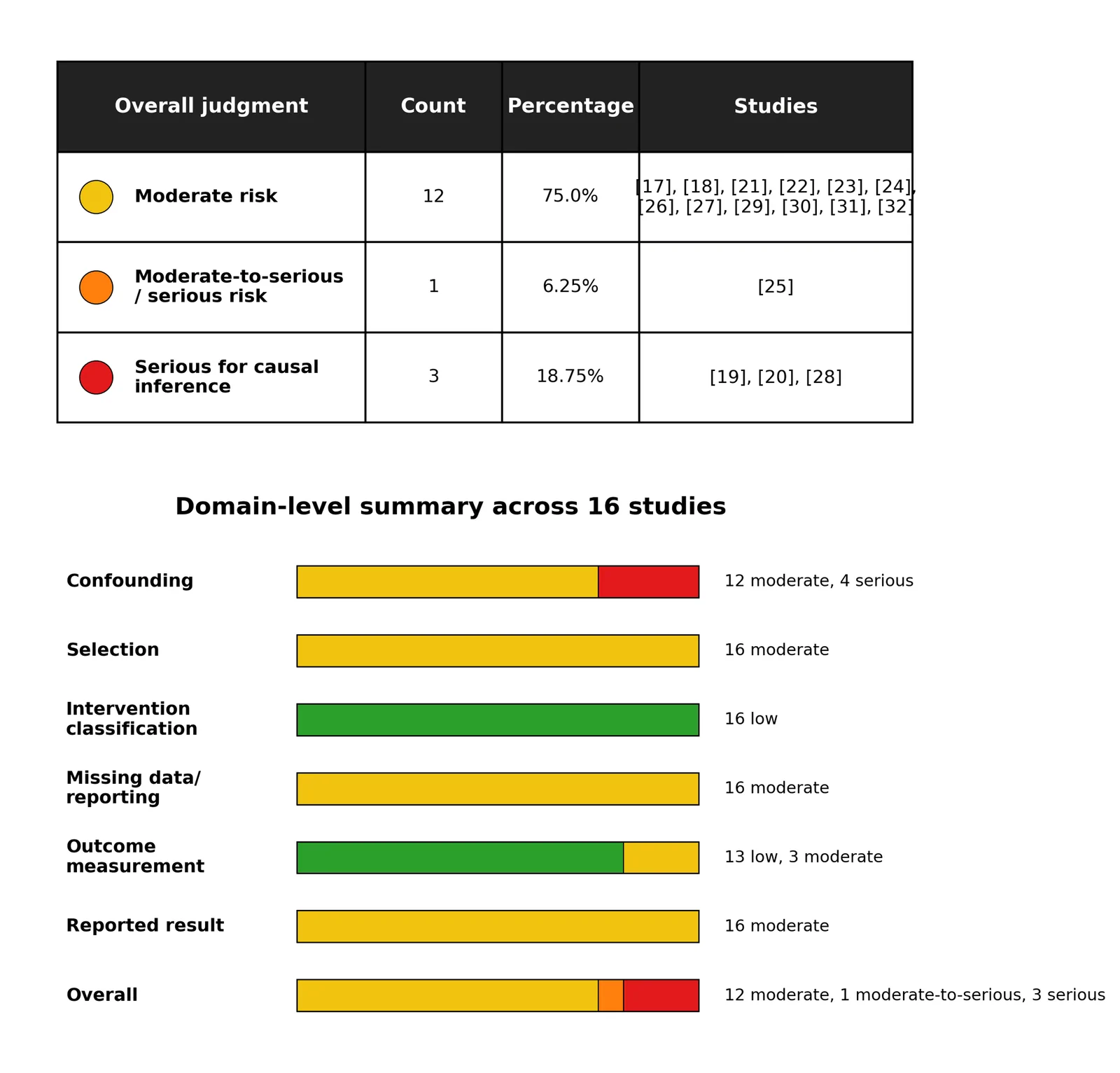
Summary traffic light map of overall risk-of-bias judgments across included studies The figure summarizes overall risk-of-bias judgments across the 16 included studies and provides a domain-level summary of judgment distribution. Twelve studies were judged as moderate risk of bias [17,18,21-24,26,27,29-32], one study was judged as moderate-to-serious risk [25], and three studies were judged as serious for causal inference because they lacked formal comparator groups [19,20,28]. Green indicates low risk, yellow indicates moderate risk or some concerns, orange-red indicates moderate-to-serious risk, and red indicates serious risk.

Taken together, the risk-of-bias assessment supports cautious interpretation of intervention effectiveness. The strongest evidence was for objective process outcomes, particularly time to analgesia and documented analgesia administration. Evidence was less certain for pain score reduction, patient satisfaction, adverse events, and emergency department length of stay because these outcomes were less consistently measured, variably reported, and less often adjusted for confounding.

Meta-Analysis

A quantitative synthesis was conducted for comparative studies reporting sufficient numerical data on time to analgesia in minutes. The effect measure was the mean difference, calculated as the intervention or protocol-driven analgesia group minus the comparator group. Therefore, negative values indicate a shorter time to analgesia and favor protocol-driven or nurse-initiated analgesia. A random-effects model was used because the included studies differed in population, intervention type, clinical setting, and comparator pathway. Because several time-to-analgesia distributions were potentially skewed, reported means were analyzed only when an accompanying variance estimate or a directly reported effect estimate with uncertainty was available; median-only data were not converted. The pooled analysis was interpreted as exploratory.

The exploratory main meta-analysis included three non-randomized comparative studies with usable effect estimates and uncertainty measures [18,25,26]. The random-effects synthesis supported shorter time to analgesia with structured pathways, with a pooled mean difference of -105.4 minutes (95% CI -164.5 to -46.3). Heterogeneity was substantial (I² = 90.4%), and the between-study variance estimate is unstable with only three studies. Because the contributing comparisons were unadjusted and non-randomized, residual confounding from baseline delay, case mix, secular trends, and clinical setting could not be excluded; the pooled estimate should therefore not be interpreted as causal. Kefyalew M et al., 2024 [25], contributed a substantially larger effect estimate than the other studies and was the major source of heterogeneity. It was retained in the primary analysis because it reported the prespecified comparative time-to-analgesia outcome with an extractable effect estimate and uncertainty measure, and its influence was examined in a sensitivity analysis. The main forest plot is shown in Figure 4.

**Figure 4:**
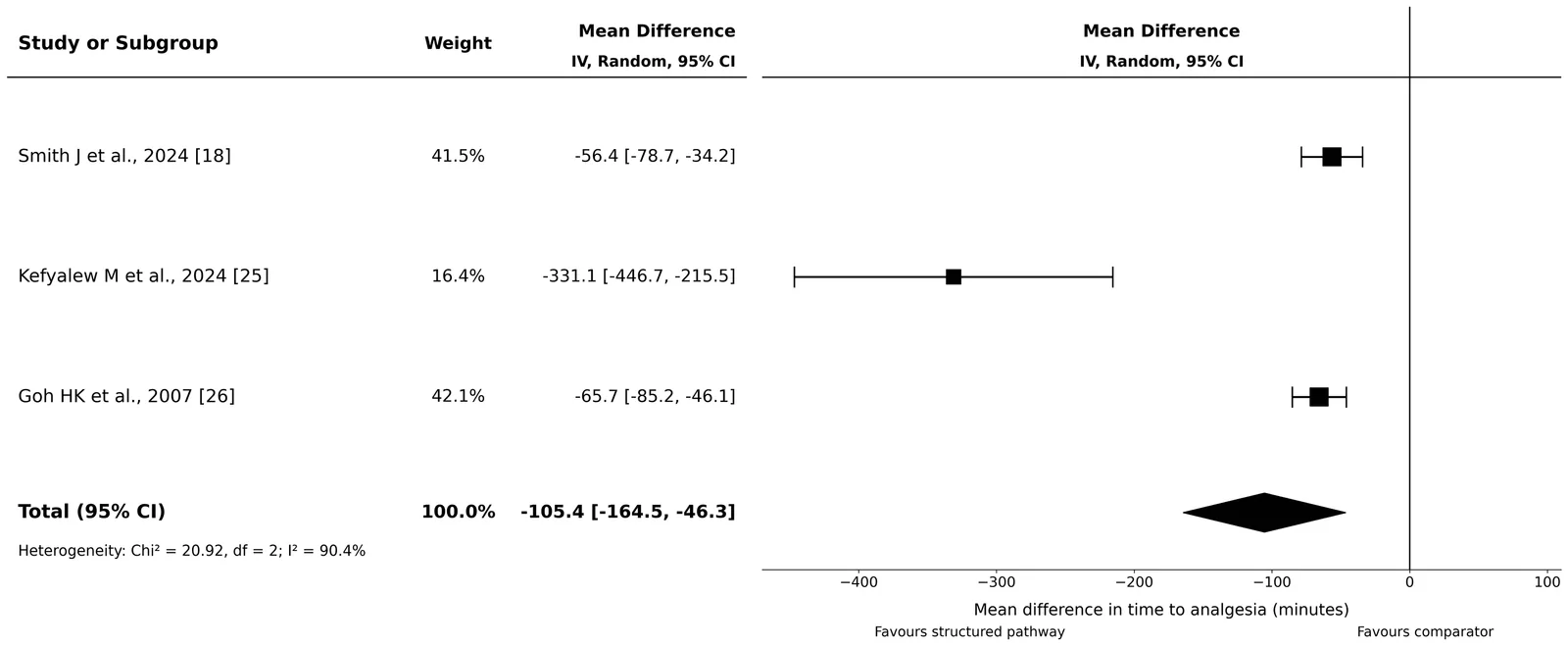
Forest plot of time to analgesia in minutes The forest plot shows the pooled mean difference in time to analgesia between protocol-driven or nurse-initiated analgesia and comparator care. Negative values favor protocol-driven or nurse-initiated analgesia. The random-effects model included Smith J et al., 2024 [18], Kefyalew M et al., 2024 [25], and Goh HK et al., 2007 [26]. The pooled effect showed a significant reduction in time to analgesia, although heterogeneity was substantial.

A sensitivity analysis was conducted excluding Kefyalew M et al., 2024 [25]. The pooled mean difference was -61.6 minutes (95% CI -76.3 to -47.0). The two remaining studies showed similar point estimates, with I² = 0.0%; however, with only two studies, this value should not be interpreted as evidence that between-study heterogeneity is absent. Publication-bias tests and prediction intervals were not performed because the number of pooled studies was too small for meaningful interpretation. The sensitivity analysis supports the direction of the association while emphasizing the uncertainty created by the small evidence base. The sensitivity forest plot is shown in Figure 5.

**Figure 5:**
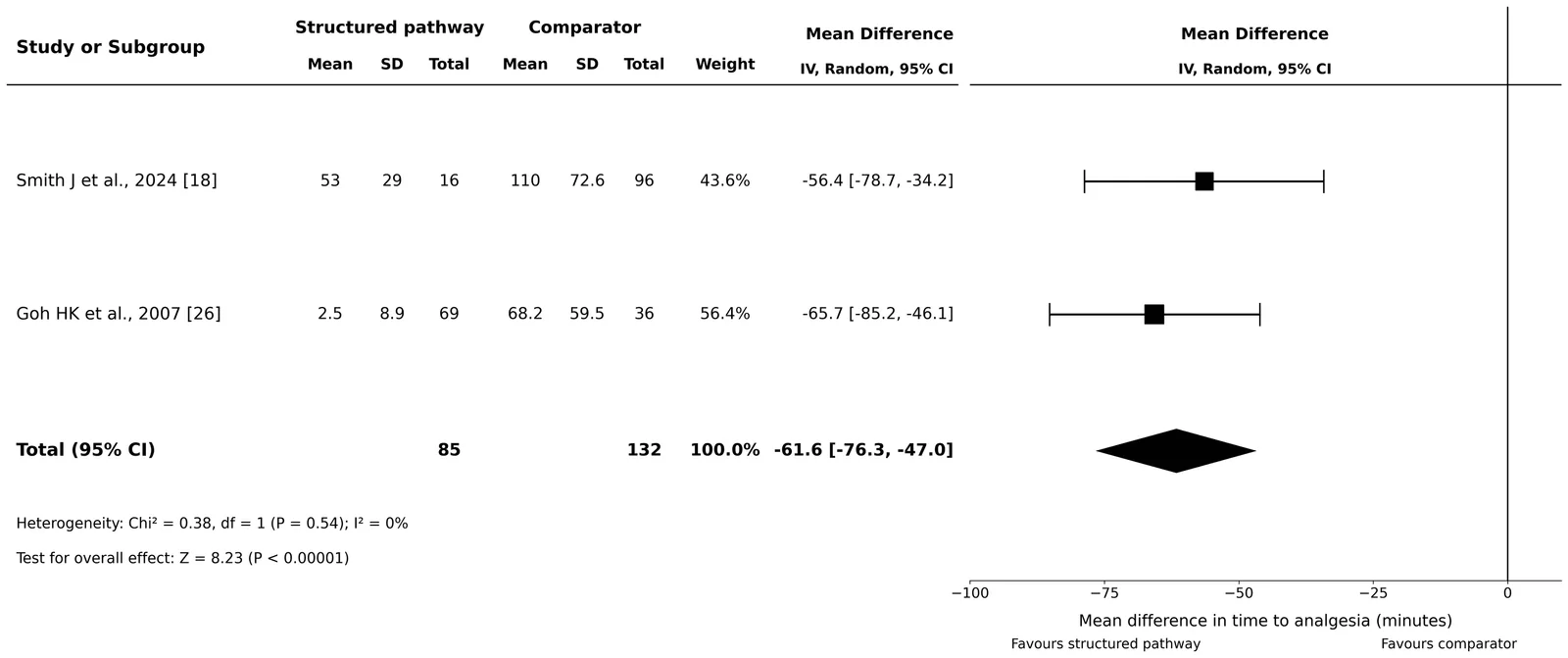
Sensitivity forest plot of time to analgesia excluding Kefyalew M et al., 2024 This sensitivity analysis excluded Kefyalew M et al., 2024, because it had a substantially larger effect estimate and contributed materially to heterogeneity in the main analysis. The two remaining studies showed similar point estimates. With only two studies, the I² estimate is imprecise and should not be interpreted as evidence that heterogeneity is absent.

Several additional studies were retained in the narrative synthesis because they reported medians, single-arm data, statistical process control outputs, or incomplete variance information [19,22,23,28,30-32]. Other comparative time-to-analgesia studies reporting means but lacking sufficient dispersion information are detailed in Table 6. These studies were not imputed into the main model because the available reports did not provide an adequately supported study-specific variance estimate. Overall, the quantitative and narrative findings generally favored earlier analgesic administration with structured pathways, but the pooled estimate should be interpreted as exploratory.

Pooling eligibility and the statistical inputs used for the quantitative synthesis are shown in Table 6.

**Table 6:** Study-by-study eligibility and statistical inputs for quantitative synthesis of time to analgesia TTA: time to analgesia; MD: mean difference; SD: standard deviation; SE: standard error; CI: confidence interval; SPC: statistical process control; QI: quality improvement; n: sample size; min: minutes. “Not pooled” indicates that the study was retained in the narrative synthesis but was not included in the quantitative meta-analysis because adequately supported variance information was unavailable or the reported statistic was not suitable for pooling.

Study	Reported TTA statistic	Variance/uncertainty	Pooling decision	Input used/reason
Smith J et al., 2024	Mean 53 vs. 110 min; reported MD -56.4	95% CI available; arm SDs available	Included	Generic inverse variance: MD -56.4; SE 11.35. Narrative arm means are rounded.
Patil A et al., 2017	Mean TTA: post-protocol 13.05 vs. pre-protocol 20.30 min	TTA SD/SE/CI not reported	Not pooled	No supported study-specific variance estimate.
Kelly AM et al., 2005	Median 31 vs. 57 min	Median/non-parametric reporting	Not pooled	Median not converted to a mean.
Woolner V et al., 2020	Mean TTA 100 vs. 129 min	No TTA SD/SE/CI; SPC/non-parametric analysis	Not pooled	Dispersion unavailable.
Kefyalew M et al., 2024	Mean 88.9 vs. 419.9 min; reported MD -331.09	95% CI available	Primary + sensitivity exclusion	Generic inverse variance: MD -331.09; SE 58.98; serious site-level confounding.
Goh HK et al., 2007	Mean ± SD 2.5 ± 8.9 vs. 68.2 ± 59.5 min	n/mean/SD available	Included	n=69 vs. 36; MD -65.7; SE 9.97; marked skewness concern.
Wong AF et al., 2025	Rooming-to-analgesia 32 vs. 50 min	SPC/QI; no conventional SE/CI	Not pooled	Retained narratively.
Barksdale AN et al., 2016	Mean 168 vs. 238 min	TTA SD/SE/CI not reported	Not pooled	Dispersion unavailable.

The certainty of evidence for the primary outcome is summarized in Table 7. Publication bias could not be meaningfully assessed because only three studies were pooled.

**Table 7:** GRADE summary of findings for the primary outcome, time to analgesia The overall certainty of evidence was rated very low because of serious risk of bias, serious inconsistency, and serious imprecision; indirectness was not considered serious, and publication bias could not be meaningfully assessed because only three studies were pooled. GRADE: Grading of Recommendations Assessment, Development and Evaluation; MD: mean difference; CI: confidence interval; I²: percentage of total variability attributable to between-study heterogeneity rather than sampling error; ED: emergency department; min: minutes.

Outcome	Evidence base	Effect estimate	GRADE domain judgments	Overall certainty	Interpretation
Time to analgesia	3 non-randomized comparative studies	MD -105.4 min (95% CI -164.5 to -46.3); exploratory	Risk of bias: serious—unadjusted non-randomized comparisons and residual confounding. Inconsistency: serious—I² = 90.4% with markedly different effect magnitude. Indirectness: not serious—ED populations and structured analgesia pathways were directly relevant. Imprecision: serious—only three studies and a wide confidence interval. Publication bias: not assessable because only three studies were pooled.	Very low	Structured pathways were associated with shorter time to analgesia, but confidence in the pooled magnitude is very limited.

Discussion

Principal Findings

This systematic review synthesized evidence from 16 studies evaluating protocol-driven, nurse-initiated, or structured analgesia pathways in emergency department settings [17-32]. Across the evidence base, the most reproducible finding was earlier analgesic administration when structured pathways allowed treatment to begin at triage or before physician assessment [18,22,25,26,32]. Quantitative synthesis supported shorter time to analgesia among studies with extractable comparative estimates [18,25,26]. Although heterogeneity was substantial in the main analysis, the sensitivity analysis continued to favor structured analgesia after removal of the most extreme effect estimate, reinforcing the direction of association rather than the precise magnitude of benefit [18,25,26].

The evidence was less uniform for downstream outcomes such as pain score reduction, patient satisfaction, adverse events, and emergency department length of stay. Several studies reported improved pain scores after protocol implementation [17,21,28], while others primarily demonstrated improvement in analgesia administration, documentation, or protocol uptake rather than consistent improvement in total length of stay [18,23,24,29,30]. This pattern suggests that protocol-driven analgesia is most strongly supported as a process-improvement intervention that reduces treatment delay, while its effect on broader emergency department flow or patient-centered outcomes may depend on local workflow, staffing, patient case mix, and the specific clinical pathway used.

Interpretation of Time-to-Analgesia Findings

Time to analgesia was the most consistent outcome, with the largest improvements generally observed where nurses were able to initiate treatment early through triage-based or nurse-led protocols [25,26]. Goh HK et al. demonstrated substantially earlier analgesia when treatment was initiated by triage nurses rather than after physician consultation [26]. Kelly AM et al. similarly reported a shorter median time to first opioid dose with nurse-initiated titrated intravenous opioid analgesia in renal and biliary colic [22]. Smith J et al. reported earlier analgesia after implementation of an intranasal fentanyl guideline for patients with hip fracture, together with improvements in selected flow outcomes [18]. Taken together, these examples support a common process effect in which structured pathways move analgesic treatment earlier in the emergency care pathway despite differences in population and intervention design.

The exploratory meta-analysis supported a statistically significant reduction in time to analgesia, but substantial heterogeneity indicates that the magnitude of the association varied considerably across settings [18,25,26]. Because the pooled studies were non-randomized and based on unadjusted comparisons, the pooled estimate should not be interpreted as a causal treatment effect. The very large effect reported by Kefyalew M et al. likely reflects both the intervention and the unusually long baseline delay in the comparator setting [25]. After excluding this study, the sensitivity analysis showed a pooled mean difference of −61.6 minutes, with a 95% confidence interval from −76.3 to −47.0 minutes, and the two remaining studies showed similar point estimates [18,26]. This supports the direction of the association while emphasizing that the precise magnitude remains uncertain.

Protocol-Driven Analgesia as a System-Level Intervention

The findings indicate that the main value of protocol-driven analgesia lies in changing the system of care rather than simply changing the analgesic drug. Many interventions acted by shifting analgesia earlier in the patient journey, particularly to triage or early nursing assessment [18,22,23,25,26,30,32]. This changes the usual workflow by allowing eligible patients to receive pre-authorized analgesia through standing orders, medical directives, or nurse-initiated protocols before physician ordering or formal physician review. By removing this sequential ordering step for appropriately selected patients, treatment can begin earlier while definitive assessment continues.

This system-level interpretation is supported by studies that showed improvements in analgesia administration and documentation even when pain outcomes were less consistently reported. Van Woerden G et al. found that revised pain guidelines increased the proportion of patients receiving analgesia, although the absolute post-implementation rate remained modest [29]. Burgess L et al. reported improvements in analgesia within 30 minutes, nurse-initiated analgesia, and pain score documentation following implementation of evidence-based pain management interventions [30]. Woolner V et al. similarly reported improved use of medical directives and reduced time to analgesia in patients with musculoskeletal pain [23]. These findings suggest that protocols improve reliability and timeliness of care, but sustained benefit may require ongoing staff engagement, audit feedback, and integration into routine emergency department workflow.

Pain Relief, Satisfaction, and Patient-Centered Outcomes

Although time to analgesia was the strongest outcome, several studies also suggested that earlier protocol-based care may improve pain relief and satisfaction. Patil A et al. reported shorter time to analgesia, greater reduction in Numeric Rating Scale scores, and higher satisfaction after implementation of a protocol-based pain management approach [21]. Butti L et al. found rapid analgesic intake and significant reduction in pain scores after a triage nursing pain protocol, although the absence of a formal comparator limits causal interpretation [28]. Santos MBD et al. reported a greater reduction in patient-reported numeric pain scores among patients receiving a nurse-initiated protocol, despite no significant reduction in total emergency department length of stay [17].

Patient satisfaction was reported less consistently and measured using different tools, which prevented quantitative synthesis. However, the available evidence suggests that patients and families may value earlier recognition and treatment of pain, particularly when analgesia is provided before prolonged waiting or diagnostic completion [20,21,25,28]. Future studies should measure patient-centered outcomes more consistently, including pain relief at standardized time points, satisfaction, acceptability, and perceived involvement in pain management decisions.

Safety Considerations

Safety findings were generally reassuring, but the evidence should be interpreted cautiously because adverse event reporting was inconsistent. Studies involving nurse-initiated opioid, ketorolac, intranasal fentanyl, or structured pediatric opioid pathways did not identify major safety concerns in the reported data [18,22,26,31]. This supports the feasibility of protocol-driven analgesia when eligibility criteria, contraindications, dosing rules, and monitoring procedures are clearly defined. However, the low number of reported adverse events may reflect limited sample size, incomplete documentation, or insufficient follow-up rather than absence of risk. Safety reporting should therefore be strengthened in future studies, especially for opioid-based protocols, older adults, patients with comorbidities, and pediatric sickle cell pain pathways.

Emergency Department Flow Outcomes

The effect of protocol-driven analgesia on emergency department flow was less consistent than its effect on time to analgesia. Smith J et al. found a shorter emergency department length of stay in hip fracture patients receiving triage-initiated intranasal fentanyl [18]. Other studies reported no clear or statistically significant length-of-stay benefit, despite improvements in analgesia delivery or pain documentation [17,24,31]. This is plausible because emergency department length of stay is influenced by many factors beyond analgesia, including imaging availability, consultation delays, bed access, admission processes, and crowding. Therefore, analgesia protocols may improve patient comfort and early care quality without necessarily shortening total emergency department stay. Flow outcomes should be interpreted as secondary system outcomes rather than direct measures of analgesic effectiveness.

Risk of Bias and Certainty of Interpretation

The evidence base was dominated by non-randomized designs, including retrospective reviews, pre-post implementation studies, quasi-experimental studies, audit/re-audit designs, quality improvement projects, and single-arm implementation studies [17-32]. No randomized controlled trial was included. Most comparative studies were judged to have a moderate risk of bias because intervention allocation was not randomized. Secular trends, staffing differences, crowding, case mix, and documentation quality may also have influenced the observed outcomes. Three studies without formal comparator groups contributed useful information regarding implementation and feasibility but were appraised separately using the JBI checklist rather than being assigned ROBINS-I overall categories [19,20,28].

Despite a generally favorable direction across different settings, countries, patient groups, and protocol types [18,22,23,25,26,30,32], the formal GRADE certainty for time to analgesia was very low because the pooled evidence was based on a small number of non-randomized studies with residual confounding, substantial heterogeneity, and imprecision. The use of objective timestamps for analgesia administration reduces concern about subjective outcome measurement but does not eliminate these design limitations. Evidence for pain intensity reduction, satisfaction, adverse events, and length of stay was also less certain because these outcomes were measured inconsistently and were less often reported with extractable variance or adjusted effect estimates.

Clinical Implications

The findings support structured analgesia pathways as a practical strategy for reducing delays in emergency department pain management, provided that their effects are interpreted within the limitations of the underlying evidence. The strongest implementation candidates are protocols with clear eligibility criteria, standardized pain scoring, medication-specific contraindications, defined escalation rules, and documentation prompts. Triage-based analgesia appears particularly valuable for painful conditions where physician assessment may be delayed, but safe initial analgesia can be protocolized, including musculoskeletal injuries, renal or biliary colic, hip fracture, and sickle cell pain episodes [18,22,23,25,26,31,32].

However, implementation should not rely on protocol availability alone. Several studies suggest that uptake depends on staff training, nursing confidence, medical governance, access to medication at triage, audit feedback, documentation tools, and ongoing multidisciplinary support [23,29,30,31]. Institutions adopting such pathways should therefore combine protocol design with implementation strategies that address workflow barriers and monitor both effectiveness and safety.

Strengths and Limitations of This Review

This review has several strengths. It focused on a clinically important and measurable emergency care problem, included diverse settings and patient populations, extracted both process and clinical outcomes, and distinguished between quantitative synthesis and narrative synthesis based on data suitability. The review also assessed risk of bias according to study design. It avoided pooling studies with incompatible or insufficiently reported outcome formats.

The review also has limitations. The included studies were heterogeneous in population, intervention type, analgesic agent, comparator pathway, and outcome measurement. Many studies were non-randomized and therefore vulnerable to confounding. Some studies reported medians, quality improvement control charts, or single-arm outcomes that could not be incorporated into the main meta-analysis. The primary meta-analysis included only a small number of studies, and the pooled estimate was affected by substantial heterogeneity. Potential skewness in time-to-analgesia distributions could not be evaluated at the participant level because the review relied on published summary statistics. The review protocol was not prospectively registered, which should also be considered when interpreting the methodological certainty of the review. Trial registries, conference abstract databases, and other grey-literature sources were not searched, so unpublished or non-indexed implementation evidence may have been missed. Some potentially relevant full texts were not retrievable. The retained review files contained aggregate reasons for the 37 full-text exclusions but not a recoverable study-level exclusion log; therefore, a report-by-report supplementary exclusion table could not be reconstructed reliably without retrospective inference. The exact executed Cochrane Library Boolean syntax and the original jamovi software version were also not preserved in the retained records. However, the final meta-analytic estimates were independently reproduced from the retained study-level inputs using a DerSimonian-Laird random-effects estimator. These limitations mean that the pooled estimate should be interpreted as exploratory and supportive rather than definitive.

Implications for Future Research

Future research should prioritize prospective comparative designs with standardized outcome reporting. At minimum, studies should report group-level sample size, mean, standard deviation, median, interquartile range, event counts, confidence intervals, and p-values for time to analgesia, pain score reduction, analgesia administration, adverse events, and emergency department length of stay. Studies should also report protocol adherence, reasons for non-use, staff training, contraindication screening, and safety monitoring. More robust comparative evaluations are needed in older adults, pediatric populations, low- and middle-income settings, and high-risk pain conditions such as sickle cell vaso-occlusive crisis and hip fracture [18,19,24,25,31].

Overall Interpretation

Overall, the findings indicate that protocol-driven and nurse-initiated analgesia pathways are associated with shorter delays to analgesia in emergency department settings. The most reliable evidence relates to time to analgesia and analgesia administration, while evidence for pain intensity, satisfaction, adverse events, and emergency department flow remains less certain. These pathways should therefore be viewed as promising system-level strategies for improving the timeliness and reliability of emergency pain care. Their implementation should be accompanied by appropriate governance, staff training, eligibility criteria, documentation standards, and safety monitoring.

## Conclusions

Protocol-driven and nurse-initiated analgesia pathways appear to improve the timeliness of analgesic administration in emergency department settings. The most consistent benefit was observed for time to analgesia, especially when eligible patients could receive analgesia at triage or before physician assessment. Evidence for pain score reduction, patient satisfaction, adverse events, and emergency department length of stay was less certain because these outcomes were measured and reported inconsistently. These findings support structured analgesia pathways as practical system-level interventions for improving emergency pain care, provided that they are implemented with clear eligibility criteria, staff training, documentation support, and safety monitoring. Given the limitations and variability of the current evidence, stronger prospective comparative studies with standardized outcome reporting are needed to better define the clinical and operational impact of these interventions.
